# Eco-friendly silver nanoparticles (AgNPs) fabricated by green synthesis using the crude extract of marine polychaete, *Marphysa moribidii*: biosynthesis, characterisation, and antibacterial applications

**DOI:** 10.1016/j.heliyon.2020.e05462

**Published:** 2020-11-12

**Authors:** Nur Syakirah Rabiha Rosman, Noor Aniza Harun, Izwandy Idris, Wan Iryani Wan Ismail

**Affiliations:** a Cell Signalling and Biotechnology Research Group (CeSBTech), Faculty of Science and Marine Environment, Universiti Malaysia Terengganu, 21030 Kuala Nerus, Terengganu, Malaysia; b Institute of Tropical Aquaculture and Fisheries (AKUATROP), Universiti Malaysia Terengganu, 21030 Kuala Nerus, Terengganu, Malaysia; c Advance NanoMaterials (ANOMA) Research Group, Faculty of Science and Marine Environment, Universiti Malaysia Terengganu, 21030 Kuala Nerus, Terengganu, Malaysia; d South China Sea Repository and Reference Centre, Institute of Oceanography and Environment (INOS), Universiti Malaysia Terengganu, Terengganu, Malaysia

**Keywords:** Materials science, Biotechnology, Nanotechnology, *Marphysa moribidii*, Silver nanoparticles, Green synthesis, Antibacterial study, Polychaetes, Nanobiotechnology

## Abstract

The non-hazardous silver nanoparticles (AgNPs) synthesised using the extract of a biological organism has gained widespread attention for various applications, mainly in healthcare. This study aimed at synthesising AgNPs using the aqueous extract of *Marphysa moribidii* (Annelida, Polychaeta) and to evaluate their antibacterial activities. AgNPs were synthesised in response to silver nitrate (AgNO_3_) with polychaete crude extract for 24 h incubation; the polychaete crude extract acted as both reducing and stabilising agents. The presence of biosynthesised AgNPs was confirmed by an analysis of colour variations from pinkish to yellowish-brown, as well as the appearance of surface Plasmon resonance (SPR) bands at 398–400 nm using ultraviolet-visible spectroscopy. Biosynthesised AgNPs were characterised by dynamic light scattering, scanning electron microscope, transmission electron microscopy, X-ray diffraction, and Fourier transform infrared spectroscopy. Biosynthesised AgNPs showed a significant effect (*p* < 0.05) on Gram-positive bacteria (*Staphylococcus aureus* and *S.epidermidi*s) and Gram-negative bacteria (*Escherichia coli*, *Klebsiella pnemoniae*, *Salmonella typhimurium*, *Serratia* sp., *Shigella sonnei*, and *Pseudomonas aeruginosa*). Thus, the crude extract of *M. moribidii* has a potential as a reducing agent for the development of future nanometal-based antibacterial agent, AgNPs, for the treatment of infectious diseases caused by pathogenic bacteria.

## 1 Introduction

Over the last several decades, the extensive production and involvement of noble metallic nanomaterials (1–100 nm) have been witnessed in several fields of study, resulting from their peculiar physical, chemical and biological properties (Syafiuddin et al., 2017). These metallic nanoparticles can be applied in diverse areas, such as electrical and electronics, beauty care products, drugs delivery, and biotechnology by incorporating their unique properties (Zhang et al., 2016; Ealias & Saravanakumar, 2017). Owing to the emergence of multidrug-resistant bacterial strains, antibacterial properties of metal nanoparticles have been thoroughly explored to combat infections of these pathogenic bacteria (Linlin et al., 2017; Baptista et al., 2018). Communicable diseases or also known as infectious diseases have been reported as the leading cause of death worldwide (Morens et al., 2004; Bloom and Cadarette, 2019). Today, the resistance of bacteria towards various antibiotics has reached a critical point, hence, discovery of a new antibacterial agent class is imperative (Jackson et al., 2018).

Nano-based antibacterial agents are gaining wide acceptance by scientific experts all over the world. Many studies have been performed on metallic nanomaterials as a potential antibacterial agent to fight various bacterial infections (Saxena et al., 2016). Nano-based antibacterial agents that either exhibit antibacterial properties of their own or improve the potency of current antibiotics are capable of preventing bacterial infections both in vitro and in vivo (Wan et al., 2016). Previously, silver (Ag) has been used as an alternate antimicrobial agent prior to the advent of silver nanoparticles (AgNPs) and has been primarily incorporated in various creams to treat infections or wounds (Saikia et al., 2015). However, somehow the effect of the Ag only lasts for a brief period of time (Méndez-Vilas, 2011). This drawback has been overcome with the utilisation of intact AgNPs with better antimicrobial properties. Interestingly, not only did AgNPs demonstrate higher lethality to microorganisms, but also lower toxicity to mammalian cells (Wan et al., 2016; Shahzad et al., 2019).

Various physical and chemical methods are typically used to synthesise AgNPs. Nevertheless, these methods are relatively expensive and have detrimental effects on the environment. Time-consuming conventional physical methods often require harsh conditions such as spacious area and high energy to produce only low yields of AgNPs, whereas chemical methods usually require toxic chemicals serve as reducing and stabilising agents, which are not ideal for biological applications (Siddiqi et al., 2018). In tandem with an increasing interest in green chemistry and sustainability, the eco-friendly biosynthesis of metal nanoparticles has drawn tremendous attention from many researchers. A variety of metal nanoparticles such as gold (Ee Pei et al., 2020), silver (Varadavenkatesan et al., 2018), iron oxide (Anchan et al., 2019) and zinc oxide (Vinayagam et al., 2019) have recently been successfully synthesised using this method. This method uses simple, cost-effective, non-toxic, and environmental-friendly protocols which mitigate negative environment impacts.

The biological approach employs naturally occurring reducing agents such as polysaccharides, enzymes, proteins and bioactive compounds of biological microorganism such as bacteria (Abo-State and Partila, 2018), fungi (Gudikandula et al., 2017), yeast (Waghmare et al., 2015), and plants (Lakshmanan et al., 2018; Shaik et al., 2018). The implementation of these biomolecules as biometrics helps to produce nanoparticles of a defined size and shape (Varadavenkatesan et al., 2018). Moreover, biomolecules play a dual role, serving as reducing agent and stabilising agents (Vinayagam et al., 2020). In contrast, the synthesis of nanoparticles using chemical methods requires separate reducing and stabilising agents. To sum up, a biological method simplifies the synthesis process and obviates the need for additional toxic reagents (Carmona et al., 2017; Yu et al., 2019). The increasing trend in the amount of papers published on the biosynthesis of nanoparticles evinces the popularity of this method.

As far as biodiversity is concerned, marine habitats are among the most diverse and richest ecosystems. Marine organisms have adapted to all sorts of harsh chemical and physical conditions, which is the primary justification for the production of many peculiar biomolecules that have never been found in terrestrial organisms. These organisms are constantly under the enormous pressure of selection, including space rivalry, predation, surface fouling and reproduction (Pathak, 2020). Marine invertebrates; polychaetes were the primary subject and focus of marine organism in this study. Marine invertebrates are a prolific source of bioactive compounds, and their biotechnological potential attracts global scientific and economic interests (Lhullier et al., 2020). Some polychaetes are utilised as baitworms, aquaculture food and an indicator for heavy metal contamination in the environment. Apart from being used as a fishing bait, the crude extract of some polychaete species containing bioactive compounds can be used to synthesise AgNPs and has potential in combating Covid-19 infection (Singh et al., 2014; Hussain et al., 2018; Masimen et al., 2020). Despite the vast number of natural products isolated from marine sources in the last decade, limited industrial applications based on marine products have so far been developed (Pathak, 2020). The aim of this study was, therefore, to investigate the potential of the marine worm *Marphysa moribidii* Idris Hutchings and Arshad, 2014 (Idris et al., 2014), polychaete species as a source of reducing and stabilising agents for the green synthesis of AgNPs with antibacterial properties.

## 2 Materials and methods

### 2.1 Materials

Silver nitrate (AgNO_3_) was purchased from Bendosen (UK). Commercial AgNPs solution and gentamycin were purchased from Sigma Aldrich (USA). *Escherichia coli*, *Klebsiella pneumoniae*, *Salmonella typhimurium*, *Serratia* sp., *Shigella sonnei*, *Pseudomonas aeruginosa*, *Staphylococcus aureus*, and *Staphylococcus epidermidis* were used as the tested bacteria in antibacterial experiments. All the pathogenic bacterial species were obtained from Microbiology Laboratory, Faculty of Science and Marine Environment, Universiti of Malaysia Terengganu (UMT). Nutrient medium (Merck, USA) and Muller-Hinton medium (Oxoid, UK) were used to grow bacteria in liquid broth culture as well as to prepare solid medium for plate culture studies by adding 2% bacteriological agar (Oxoid, UK). All the glassware and media used for antibacterial assay were sterilised in an autoclave at 121 °C. 3–(4,5–dimethylthiazol–2–yl)–2,5 diphenyltetrazolium bromide (MTT) used for visualising the bacteria growth was purchased from Abcam (USA).

### 2.2 Preparation of *M. moribidii* crude extract

Polychaetes were collected during low tide from west coast of peninsular Malaysia. The sediment and other adherent materials were entirely removed by washing thoroughly with tap water and rinsing with ddH_2_O. Polychaetes were crushed and added to 100 mL ddH_2_O for the preparation of the crude extract. The mixture was incubated on the bench for 1 h before filtration with Whatman No.1 filter paper . The aqueous crude extract was stored in a refrigerator for further use (Rosman et al., 2020).

### 2.3 Biosynthesis of AgNPs

**Figure 1:**
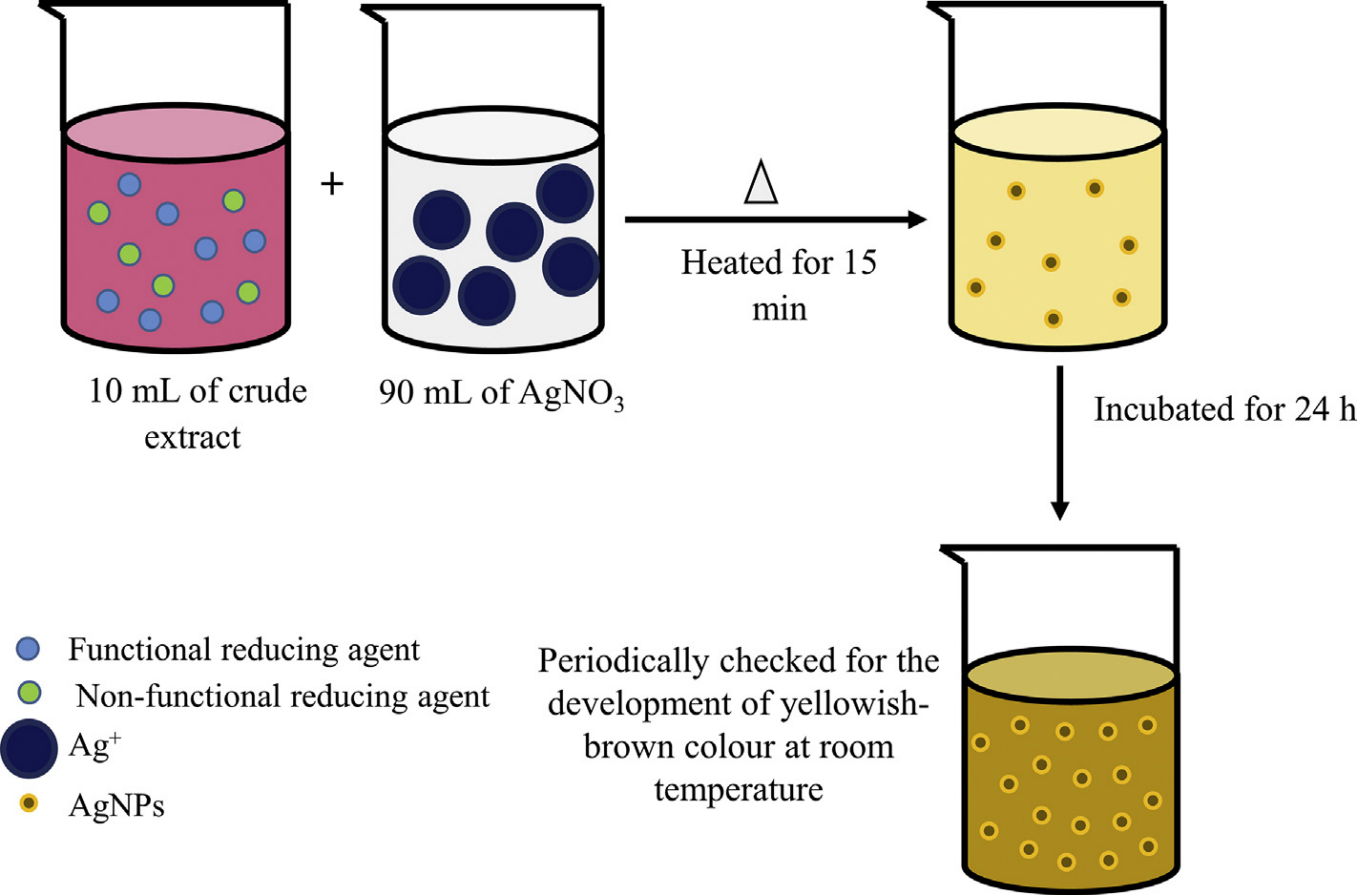
Synthesis of AgNPs from the crude extract of *M. moribidii*. Functional reducing agent is the one that is ready to convert AgNO_3_ to AgNPs, and a non-functional reducing agent is the one that is not active to convert AgNO_3_ to AgNPs due to the presence of ions, which neutralise the functional group of reducing agent. The pale yellowish colour of the solution indicates the low formation of AgNPs, while the yellowish-brown colour indicates the high formation of AgNPs.

Biosynthesis of AgNPs has been completed, as shown in Figure 1. A total of 10 mL of aqueous crude extract was added to 90 mL of AgNO_3_ solution. After 24 h of incubation, the samples were kept at room temperature (30 °C ± 2) and periodically checked for the development of yellowish-brown colour in the solution indicating the formation of AgNPs. Two types of negative controls were developed by mixing 10 mL of ddH_2_O with 90 mL of 1 mM AgNO_3_ solution. Positive control commercial AgNPs were purchased from Sigma Aldrich. The experiment was performed in triplicate. Colour variations of the reactions mixture from pinkish to yellowish-brown were recorded through visual observation.

### 2.4 Characterisation of biosynthesised AgNPs

#### 2.4.1 UV-Vis spectrophotometry

The bio-reduction of Ag^+^ in aqueous solution was then monitored by measuring the surface Plasmon resonance (SPR) spectrum of the solution within a range of 300–600 nm using the UV-Vis spectrophotometer (UV 1800, Shimadzu, Japan). Aqueous AgNO_3_ (1 mM) was used as a blank. The UV-Vis spectra were obtained from automated software UV Probe.

#### 2.4.2 Dynamic light scattering (DLS)

The DLS was carried out in a computer-controlled particle size analyser, ZETA Sizers Nanoseries (Malvern Instruments Nano ZS, Malvern Analytical, USA) at 25 ± 0.1 °C with a scattering angle of 90°. Prior to the experiment, AgNPs were filtered using a 0.2 μm syringe filter to remove unwanted debris such as dead cells. Biosynthesised AgNPs were then dispersed in water and sonicated using an ultrasonic water bath for 10 min to separate the aggregated AgNPs.

#### 2.4.3 Scanning electron microscope (SEM)

The SEM JSM-6390 LA (JEOL, USA) was operated at an accelerating voltage of 15–20 kV. All samples were prepared by a mounting process in which a drop of AgNPs was deposited onto dry poly-L-lysine and left overnight. The samples were then coated with gold by means of a sputter coating (Auto Fine Coater-JEOL) before being transferred and observed under the microscope.

#### 2.4.4 Transmission electron microscope (TEM)

TEM was operated at ambient temperature using Tecnai G2 Spirit Biotwin (FEI Company, USA) at a voltage of 100–200 kV to analyse the shape and size of biosynthesised AgNPs. Samples were prepared by drop-casting of diluted biosynthesised AgNPs on a coated copper grid. Results were analysed using ImageJ software to predict the size distribution.

#### 2.4.5 Fourier-transform infrared (FTIR) spectroscopy

The chemical composition of polychaete crude extract was determined using Perkin Elmer of Spectrum 100. A total of 40 ml of crude extract was freeze-dried and prepared using KBr disk (3% of KBr). The IR absorbance of samples was measured using the Nicolet 520 FTIR spectrophotometer. All measurements were carried out in a range of 400–4000 cm^−1^ at a resolution of 4 cm^−1^.

#### 2.4.6 X-ray diffraction (XRD) analysis

The XRD was performed to detect the presence of Ag in the polychaete crude extract that c be collected from the environmental contaminant. X-ray diffractometer operated at the voltage of 30 kV and a current of 30 mA was used to identify the phase of the polychaete crude extract. This test was analysed by the X-ray diffractometer MiniFlesx II diffractometer (Rigaku, Japan) equipped with X'celerator using Cu Kα radiation in a range between 0 and 110° (2θ).

### 2.5 Screening of the antibacterial efficacy of AgNPs against multiple bacteria caused diseases

#### 2.5.1 Preparation of turbidity standard for bacterial inoculum

Bacteria suspension was prepared from fresh bacteria colonies by re-suspending them in the Mueller-Hinton broth (MHB) and measured by spectrophotometer at a wavelength of 550 nm. Dilution was performed to obtain a reading 0.125, which is equivalent to the 0.5 McFarland turbidity standard (Wardle et al., 1995).

#### 2.5.2 Disk diffusion method

Disk diffusion method was performed to screen the antibacterial activity of biosynthesised AgNPs according to the Kirby-Bauer disk diffusion method (Jorgensen and Turnidge, 2015). Prior to the experiment, pure cultures of eight human pathogens, namely, *E. coli*, *K. pneumoniae*, *S. typhimurium*, *Serratia* sp., *S. sonnei*, *P. aeruginosa*, *S. aureus*, and *S. epidermidis* were subcultured on nutrient agar (NA) and incubated at 37 °C overnight. The dry surface of the Mueller-Hinton agar (MHA) plates was inoculated with pathogenic bacterial strains (0.5 McFarland) by swabbing over the entire sterile agar surface. Sterile paper disk (Whatman filter paper No.1) was loaded with 20 μL biosynthesised AgNPs and compared to standard antibiotic gentamycin (1 mg/mL) as a positive control while crude extract and ddH_2_O were negative controls.

#### 2.5.3 Well dilution method

Well dilution method was conducted according to the method recommended by CLSI protocol with some modifications (Jorgensen and Turnidge, 2015; Balouiri et al., 2016). Biosynthesised AgNPs were prepared by two-fold serial dilution, with a maximum volume per well of 100 μL. Growth control (bacterial suspension without AgNPs), a positive control (gentamycin), negative controls (crude extract and ddH_2_O), and sterility control (MHB without bacterial suspension and AgNPs) were also prepared. 100 μL of the bacterial suspension adjusted to the 0.5 McFarland standard was added to 96-well plate except for the well for a sterility control. The plate was incubated at 37 °C for 18–24 h. After incubation, 20 μL of 5 mg/mL MTT was added to each well. The formation of purple-blue formazan indicated a bacteria growth (Grela et al., 2018).

### 2.6 Statistical analysis

Each experiment was at least performed triplicate. All data shown in the table and figure were quantified using Microsoft Excel, ImageJ, and software Origin Pro 9. Statistical significance of the experimental results (significance level of *p* < 0.05) was calculated using IBM SPSS V.22 (IBM Corporation, Endicott, NY, USA) using one-way ANOVA and Tukey's post- hoc test.

## 3 Results and discussion

### 3.1 Biosynthesis of AgNPs

**Figure 2:**
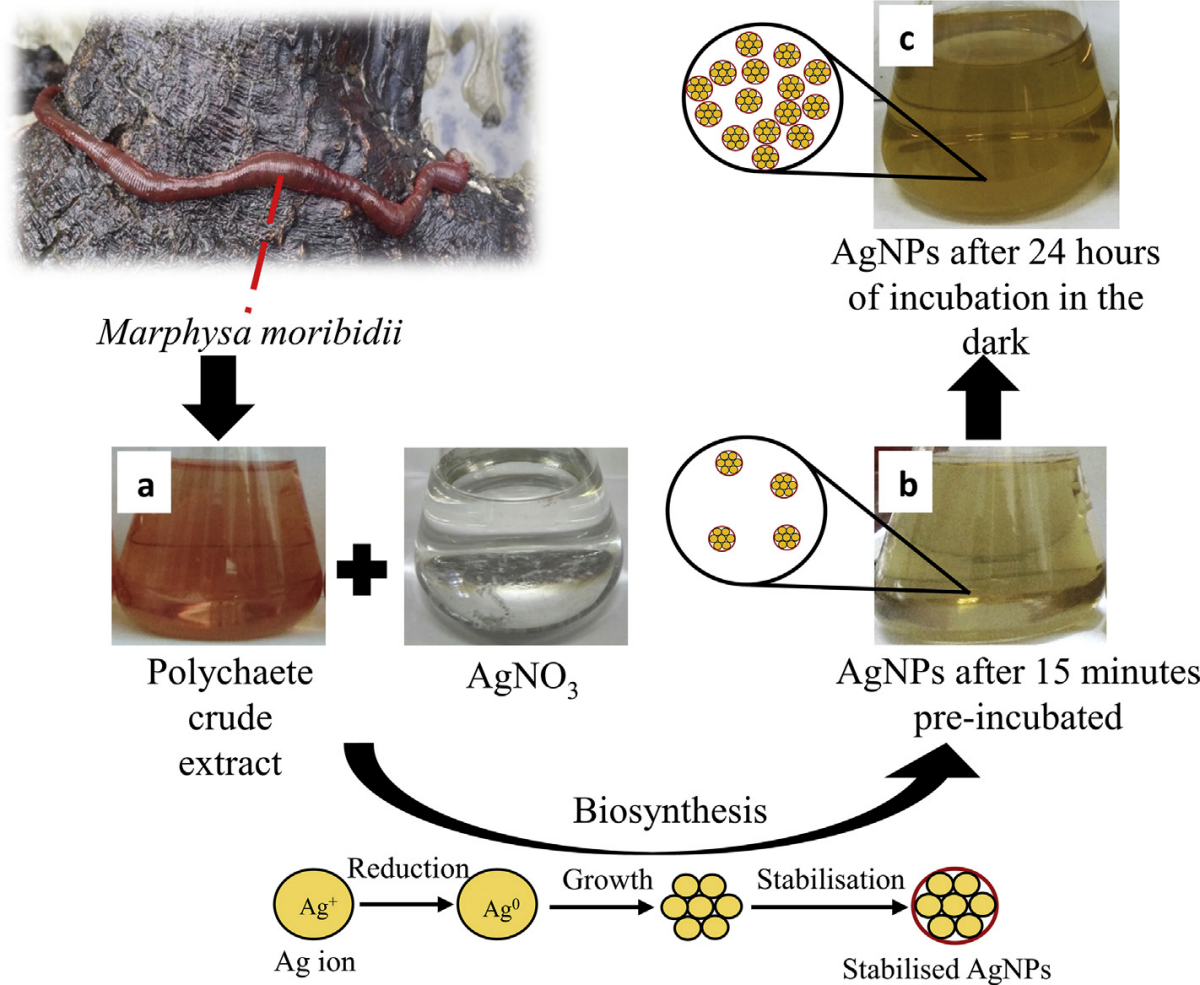
Mechanism of the AgNPs formation. The colour of (a) polychaete crude extract, (b) polychaete crude extracts with AgNO_3_ immediately after 15 min of the pre-incubation period, and (c) AgNPs after 24 h of incubation in the dark.

This study described the biosynthesis of AgNPs using an aqueous extract of *M. moribidii* with an addition of AgNO_3_. Change in the colour of solution from pinkish (Figure 2(a)) to yellowish (Figure 2(b)) after addition of polychaete crude extracts with AgNO_3_ was observed immediately after 15 min during the pre-incubation period. The colour was fully yellowish-brown (Figure 2(c)) after 24 h of incubation. The colour changes in the AgNPs solution was attributed to the excitation of surface Plasmon resonance (SPR), which indicates that bio-reduction of Ag^+^ to AgNPs has confirmed the biosynthesis of AgNPs (Ayad et al., 2018). Reduction of Ag^+^ to AgNPs by the crude extract of *M. moribidii* occurred faster than studies of AgNPs synthesis using the crude extract of different polychaete species by Singh et al. (2014) and Hussain et al. (2018) which required two hours and two weeks of incubation, respectively, to allow the reduction to occur.

### 3.2 Characterisation of biosynthesised AgNPs

**Figure 3:**
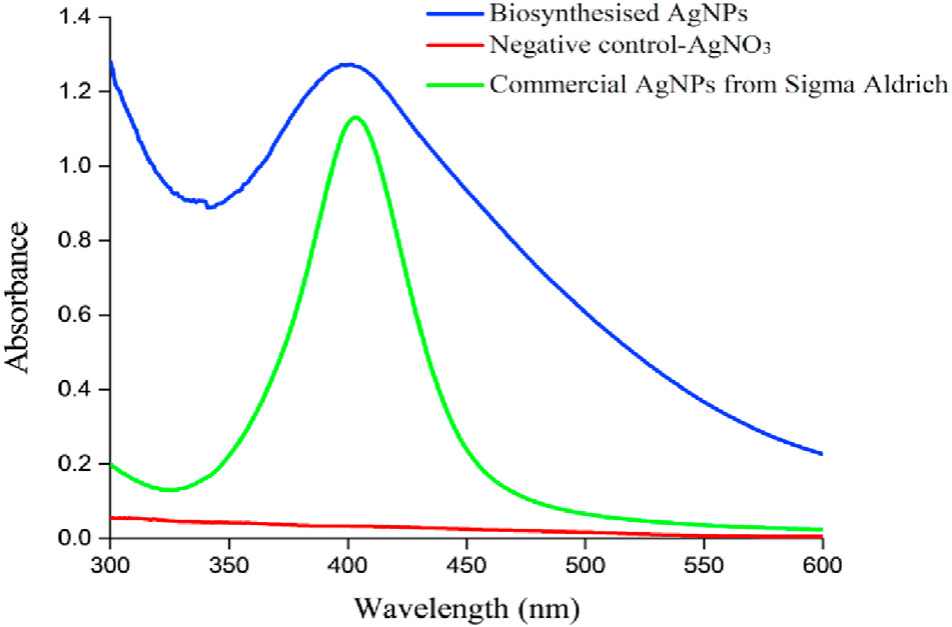
UV-Vis absorption spectra of biosynthesised AgNPs, a negative control of AgNO_3_ solution and commercial AgNPs (positive controls) by Sigma Aldrich.

Noble metals are known to exhibit unique optical properties due to the property of SPR (Yadav et al., 2018). The optical properties of biosynthesised AgNPs were assessed by measuring the absorption spectrum using the UV-Vis spectrophotometer over a wavelength range of 300–600 nm, as shown in Figure 3. A strong broad absorption peak was observed at 398 nm–400 nm, which attributed to the SPR of the AgNPs. This broad absorption peak implied that the size of biosynthesised AgNPs was larger, while the presence of SPR peak at 380 nm–450 nm indicated the formation of AgNPs (Ndikau et al., 2017). According to the literature, the absorption band located in a range of 390 nm–420 nm in the UV–Vis spectra might be attributed to the spherical-shaped AgNPs (Chowdhury et al., 2016; Vinayagam et al., 2018). The results were compared to the other studies using different polychaete species, in which Singh et al. (2014) and Hussain et al. (2018) recorded an absorption spectrum of AgNPs of 418 nm–420 nm and 411 nm, respectively. The discrepancy in these results might be due to a particle size variance. It is well established that the SPR bands are very sensitive and highly dependent on the nanoparticles size, shape, AgNO_3_ concentration and the type of biomolecules presented in the biological extract (Raja et al., 2017; Ruttkay-nedecky et al., 2019).

**Figure 4:**
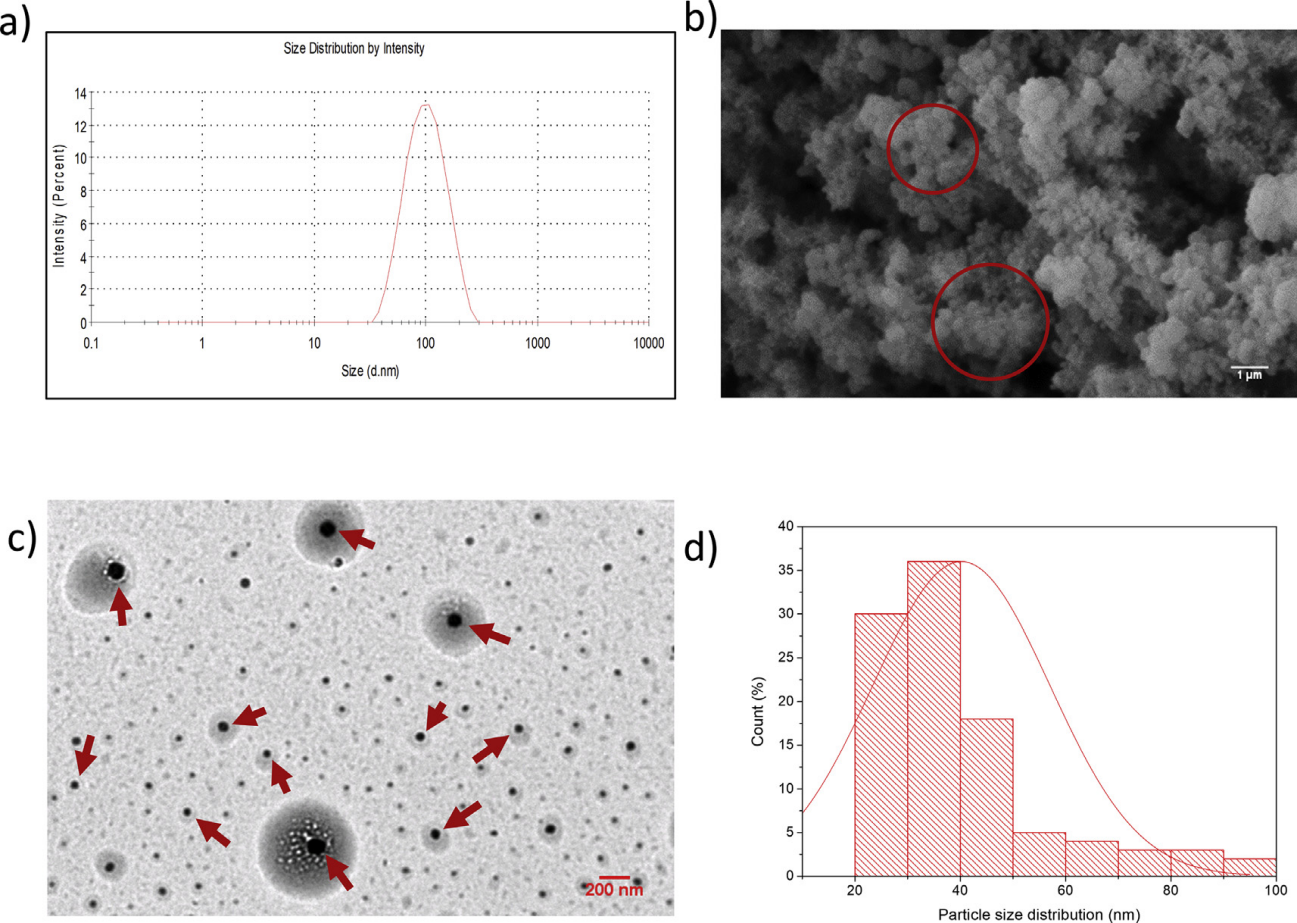
Characterisation of biosynthesised AgNPs via (a) dynamic light scattering (DLS), (b) scanning electron microscopy (SEM) and (c) transmission electron microscopy (TEM). (d) The particle size distribution of biosynthesised AgNPs obtained via TEM.

Three different techniques (DLS, SEM and TEM) were employed to determine the size and shape of biosynthesised AgNPs. The DLS was first used to predict the size of biosynthesised AgNPs. The results showed that the biosynthesised AgNPs were 91.21 nm in size with a PDI value of 0.163, indicating a moderate polydispersity sample (Figure 4(a)). However, DLS measurement is considered to be more sensitive to larger particles (aggregated AgNPs) because they tend to cover the smaller ones. The DLS principle considers a solvent layer around the particle to be a part of it. Thus, the results from DLS may not be precise compared to SEM or TEM. The SEM image revealed that the observed biosynthesised AgNPs were almost spherical (Figure 4(b) in red circle). However, the formation of biosynthesised AgNPs was poorly dispersed, and the aggregation of particles could be observed. Due to the fact that the smaller size of biosynthesised AgNPs could not be viewed under a lower resolution of SEM, the TEM with a higher magnification was further used to validate the shape and size of the biosynthesised AgNPs. The results of TEM revealed that the biosynthesised AgNPs were well dispersed and in spherical-like in shape (Figure 4(c)). The biosynthesised AgNPs were in a range between 20 nm and 100 nm with an average particles size of 40.19 nm (Figure 4(d)). This result was contradicted with the finding obtained by Singh et al. (2014) which showed that the biosynthesised AgNPs were relatively spherical and triangular in shape, and ranged in size from 40 nm to 90 nm. The discrepancies might be due to the variation of reducing and stabilising agents of the different species of polychaetes.

**Figure 5:**
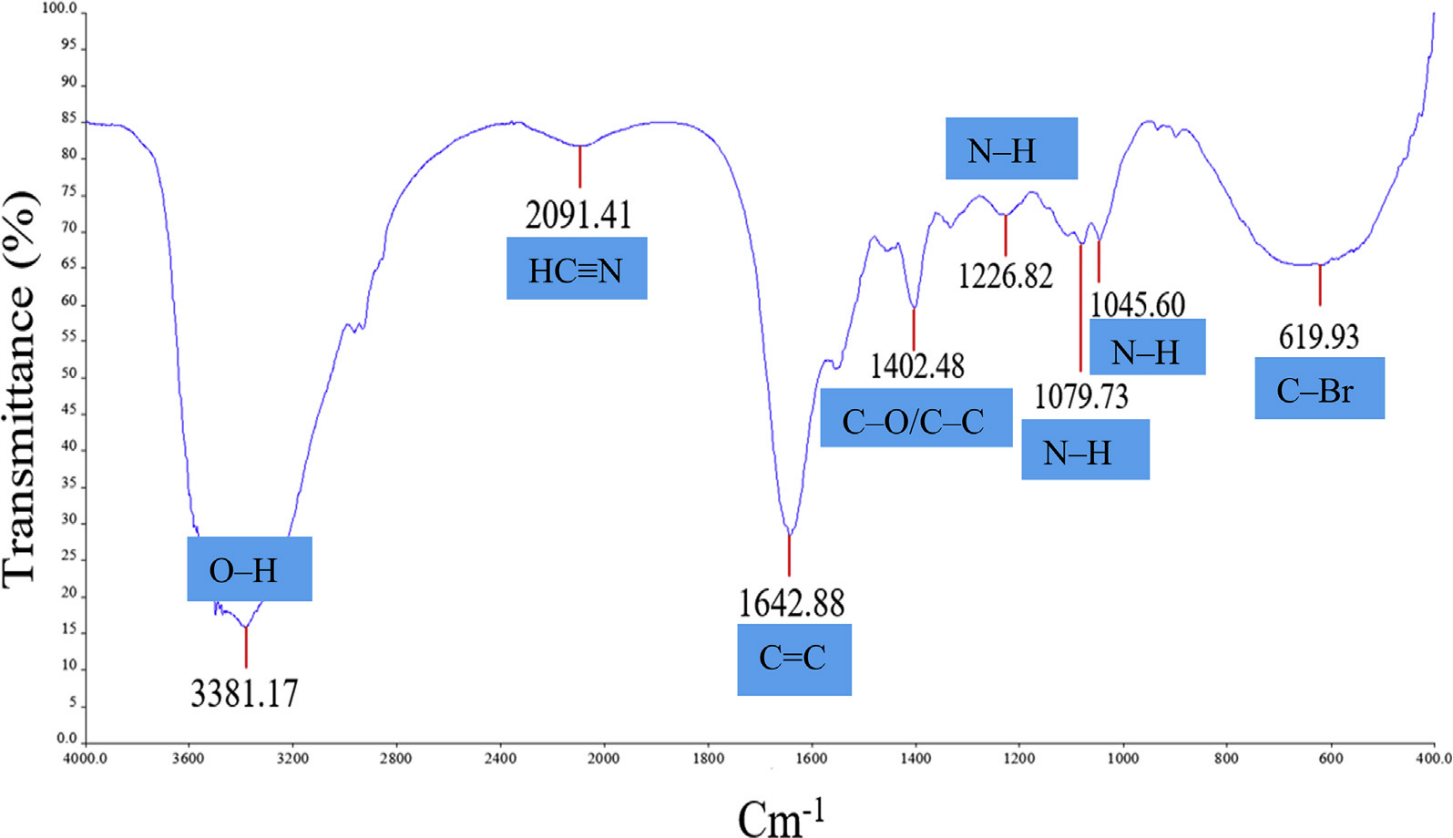
FTIR spectrum representation of *M. moribidii* crude extract.

The FTIR was used to identify the potential reducing and stabilising agents responsible for the biosynthesis of AgNPs. The functional groups in the crude extract of polychaete were found to be peaks in Figure 5. Absorption bands at 3381.17 cm^−1^ belong to the stretching of (O–H) group in carboxylic acids and phenols which possibly found in the biomolecules of polychaete such as ethers, sterols, carbohydrates and proteins. The broad peak at 2091.41 cm^−1^ was attributed to nitrile (HC≡N) stretching. The peak at 1642.89 cm^−1^ was due to *ν*(C=C) stretching of alkenes group which exist in proteins. The peak at 1402.48 cm^−1^ was due to *ν*(C–O) group in carboxylic acids or *ν*(C–C) group in aromatics which commonly found in amino acids, fatty acids, and other biomolecules. In addition, the bands at 1226.82 cm^−1^, 1079.73 cm^−1^ and 1045.60 cm^−1^ can be attributed to (N–H) stretching in primary and secondary amines of amino acids, peptides and proteins. Moreover, the band at 619.93 cm^−1^ belongs to *ν*(C–Br) group of alkyl halides.

Although extensive studies have been conducted on the biosynthesis of AgNPs, the exact mechanisms involved in these processes remain unclear. However, it has been proposed that the synthesis and stabilisation of AgNPs are probably to be achieved by organic compounds such as carboxylic acids, phenols, alkene, amine, and aromatic rings which are the main components of amino acids, proteins, fatty acids, ether, and carbohydrates. It is suggested that these functional groups could have reacted with Ag^+^ to produce AgNPs and subsequently capped around AgNPs as reported in other studies (Singh et al., 2014; Mohammadlou et al., 2016; Bhatnagar et al., 2019). The presence of (O–H) groups in the phenolic compound or carboxylic acid was known to induce and stabilise the reduction of AgNO_3_ to AgNPs. The phenolic-OH oxidised to its quinone form and produced electrons for reduction of Ag^+^, whereas the carboxylic group was bound to the surface and capped around the synthesised AgNPs (Farrokhnia et al., 2019; Hamouda et al., 2019). The functional groups such as alkene, amine, and aromatic rings are also the component of enzyme, and protein which have been speculated to be involved in the synthesis and prevention of agglomeration of AgNPs (Mohammadlou et al., 2016; Rashid et al., 2017; Ahmad et al., 2019).

**Figure 6:**
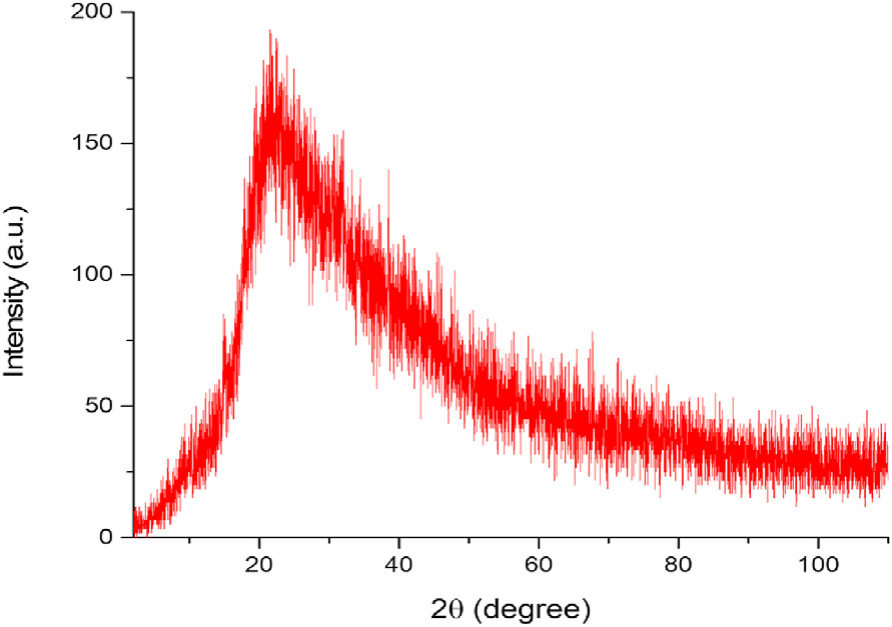
XRD patterns for *M. moribidii* crude extract.

XRD is a rapid analytical technique used to investigate the Ag metal contamination in the polychaete crude extract that might be existed in the surrounding environment (sampling site). The amorphous phase of the polychaete crude extract was demonstrated by the presence of a broad hump with a high–intensity peak around 2θ = 21.68° and no sharp diffraction peaks, as predicted for crude extract (Figure 6). The XRD pattern showed no sharp diffraction peaks in the whole spectrum of 2θ with values ranging from 0 to 110, indicating no presence of contaminant Ag in the polychaete crude extract. Therefore, this study has confirmed that AgNPs were not originated from the body of polychaetes itself, but were successfully reduced using polychaetes extract with AgNO_3_.

### 3.3 Assessment of antibacterial activity of biosynthesised AgNPs

Antimicrobial effects of the synthesised AgNPs were evaluated against eight human pathogens (Table 1) using a disk diffusion method. The method evaluated the inhibitory zones, which depended on how effective AgNPs were in stopping the growth of the bacterium. According to the results of disk diffusion method, the maximum antibacterial activity was observed against *S. epidermidis*, which showed a 9.18 ± 0.12 mm zone of inhibition followed by *P. aeruginosa* (8.94 ± 0.15 mm), *S. aureus* (8.54 ± 0.13 mm), *E. coli* (8.39 ± 0.12 mm), *S. typhimurium* (8.13 ± 0.20 mm), *S. sonnei* (7.77 ± 0.19 mm), and *Serratia* sp. (7.98 ± 0.11 mm). The lowest was observed against *K. pnemoniae* (6 mm). These results were significantly different from a negative control (*p* < 0.05). However, it was quite apparent that a positive control (gentamycin) showed a better inhibition zone ranging from 22.78 ± 0.25 mm to 31.66 ± 0.18 mm. The Kirby-Bauer disk diffusion method relies on the diffusion of the test substance from a sterile paper disk to bacterial cultures. Thus, this result might be affected by the diffusion rate of particles as metal nanoparticles have low diffusion rate as compared to standard antibiotics (Kourmouli et al., 2018). As a consequence, metal nanoparticles do not travel far from deposition disks to interact physically with the bacterial cells.

**Table 1:** Antibacterial activity of biosynthesised AgNPs on selected bacteria via a disk diffusion method. The average values of the three calculations were presented as mean ± SEM (standard error of the mean).

Bacteria	Inhibition zone (mm)			
	Biosynthesised AgNPs	Positive control	Negative control	
		Gentamycin	Crude extract	ddH_2_O
*E.coli*	8.39 ± 0.12^a^^,^^b^	28.13 ± 0.17	0	0
*K. pnemoniae*	6.00 ± 0.00^a^^,^^b^	22.78 ± 0.25	0	0
*S. typhimurium*	8.13 ± 0.20^a^^,^^b^	23.79 ± 0.29	0	0
*Serratia* sp.	7.98 ± 0.11^a^^,^^b^	26.15 ± 0.27	0	0
*S. sonnei*	7.77 ± 0.19^a^^,^^b^	23.79 ± 0.26	0	0
*P. aeruginosa*	8.94 ± 0.15^a^^,^^b^	31.66 ± 0.18	0	0
*S. aureus*	8.54 ± 0.13^a^^,^^b^	27.00 ± 0.20	0	0
*S. epidermidis*	9.18 ± 0.12^a^^,^^b^	31.40 ± 0.24	0	0

a Significantly different when compared to crude extract, *p* < 0.05.

b Significantly different when compared to ddH_2_O, *p* < 0.05.

**Figure 7:**
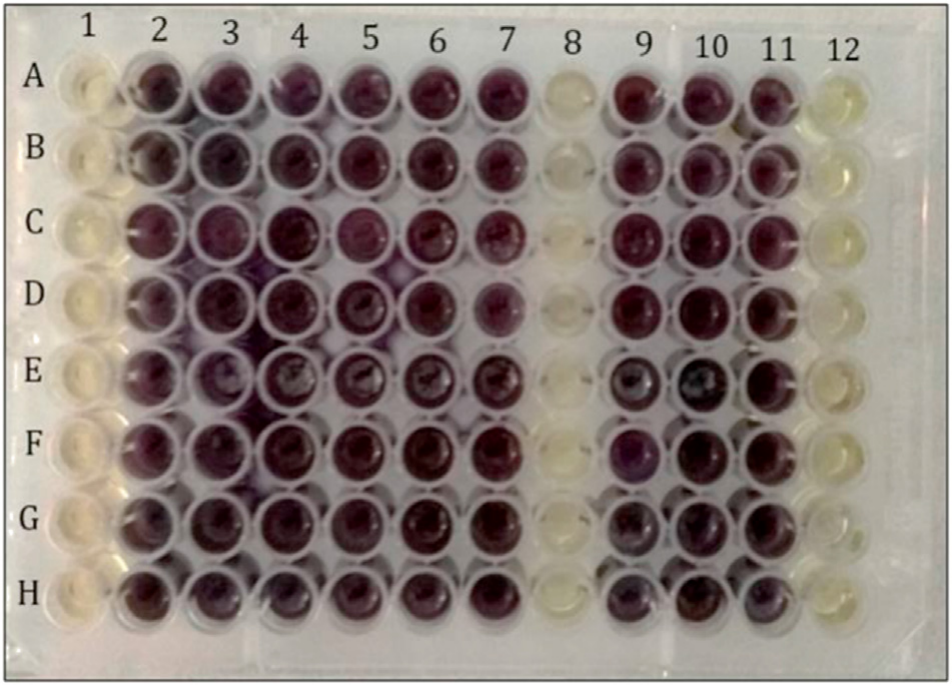
Antibacterial activities of biosynthesised AgNPs on selected bacteria via well dilution method. (A) *E. coli*, (B) *K. pneumoniae*, (C) *S. typhimurium*, (D) *Serratia* sp., (E) *S. sonnei*, (F) *P. aeruginosa*, (G) *S. aureus*, and (H) *S. epidermidis*. (1) 100%, (2) 50%, (3) 25%, (4) 12.5%, (5) 6.25%, (6), 3.125%, (7) growth control, (8) gentamycin, (9) AgNPs Sigma, (10) crude extract, (11) ddH_2_O, and (12) Sterility control. All the bacteria were inhibited at 100% concentration (stock solution) of biosynthesised AgNPs. The concentration of biosynthesised AgNPs was prepared using two-fold serial dilution from the stock solution.

Well dilution method was carried out to overcome this problem, as biosynthesised AgNPs can directly interact with pathogenic bacteria. Figure 7 shows the results of a well diffusion method of biosynthesised AgNPs. All the bacteria tested were inhibited at 100% concentration (stock solution) of biosynthesised AgNPs. However, the exact concentration of the stock solution cannot be determined due to the oxidation of biosynthesised AgNPs during the purification step, which is one of the limitations of this study.

Pathogenic bacteria are a well-known origin of infectious disease. They can be transmitted from one person to another, which can cause death to humans if not treated early, especially those with a weak immune system (Bloom and Cadarette, 2019). Most bacterial infections can be treated with antibiotics. However, the long-term use of antibiotics promotes the development of antibiotic-resistance (Ventola, 2015). Antibiotics remove drug-sensitive competitors, while resistant bacteria reproduce and grow as a result of natural selection (Read and Woods, 2014). Besides, susceptible bacteria could gain resistance through genetic mutations or may acquire DNA that encodes resistance properties from other resistant bacteria (Bennett, 2008). AgNPs, as a result, can be used as an effective antibacterial tool to prevent the development of bacterial resistance. AgNPs are potentially to be used as broad-spectrum antibacterial agents against Gram-negative, Gram-positive and multidrug-resistant bacterial due to their smaller size and high surface charge (Baptista et al., 2018).

**Figure 8:**
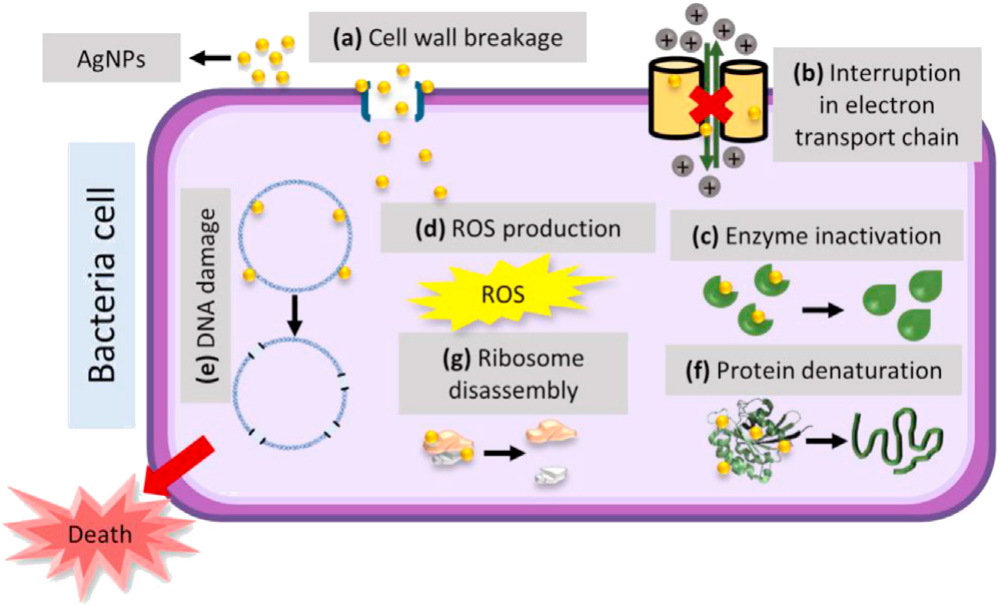
Antibacterial mechanism of AgNPs on bacteria. Interaction of AgNPs with microbial cells can cause; a) cell wall breakage, b) interruption in the electron transport chain, c) enzyme inactivation, d) generation of reactive oxygen species (ROS), e) DNA damage, f) protein denaturation, and g) ribosome disassembly. These interactions can lead to cell death.

Even though the usage of AgNPs are widespread in many antibacterial applications, the mechanism of action on microbes is still obscure. There are, however, various theories on the action of AgNPs on microbes to generate the bactericidal effect, as presented in Figure 8. AgNPs can penetrate through cells by attaching themselves to bacteria membranes, and altering the negative charge of cell walls to make them more permeable (Rizzello and Pompa, 2014). It can induce structural alterations in proteins that lead to cell destruction (Yuan et al., 2017). Moreover, the release of Ag^+^ by AgNPs will interact with and inactivate thiol groups of many vital enzymes (Slavin et al., 2017). This problem can also lead to the generation of reactive oxygen species (ROS) due to inhibition of a respiratory enzyme by Ag^+^, which can attack the cell itself (Slavin et al., 2017). Not just that, AgNPs can also alter the phosphotyrosine profile of microbial peptides by dephosphorylating the peptide substrates on tyrosine residues, which leads to inhibition of signal transduction and stop the growth (Dakal et al., 2016; Krishnanand and Sekhar, 2018). Lastly, AgNPs can interact with ribosomes, contributing to their denaturation, which can cause inhibition of translation and protein synthesis (Roy et al., 2019).

## 4 Conclusions

This study presented a facile, non-toxic and eco-friendly biosynthesis of AgNPs using aqueous extract of polychaete, *Marphysa moribidii*. The biomolecules in the polychaete extract reduced the AgNO_3_ to form AgNPs in a shorter period, hence may be used as an alternative to current conventional methods. AgNPs have been characterised using various methods, and their antibacterial activity has been tested against pathogenic bacteria. The biosynthesis of AgNPs was confirmed through changes in colour from pinkish to yellowish-brown and formation of SPR peak at 398 nm–400 nm. Biosynthesised AgNPs were found to be spherical with an average size of 40.19 nm. The presence of functional groups such as carboxylic acids, phenols, alkene, amine, and aromatic in the polychaete crude extract was likely to be the main component of reducing and stabilising agents in AgNPs synthesis. Moreover, biosynthesised AgNPs have demonstrated a significant positive antimicrobial effect on *E. coli*, *K. pneumoniae*, *S. typhimurium*, *Serratia* sp., *S. sonnei*, *P. aeruginosa*, *S. aureus*, and *S. epidermidis*. Biosynthesised AgNPs could be a promising alternative of nano-based antibacterial agent for fighting various pathogens and could also scale up economic viability in the future. Nevertheless, these findings should be further investigated in order to improve the long-term stability of biosynthesised AgNPs especially the capping effect of biomolecules. Besides, a detailed study is necessary to validate the toxicity reaction and mechanism of action of AgNPs to make the approach economically more competitive and sustainable.

## Declarations

### Author contribution statement

Nur Syakirah Rabiha Rosman: Conceived and designed the experiments; Performed the experiments; Analyzed and interpreted the data; Wrote the paper.

Noor Aniza Harun: Conceived and designed the experiments; Analyzed and interpreted the data; Wrote the paper.

Izwandy Idris: Conceived and designed the experiments; Wrote the paper.

Wan Iryani Wan Ismail: Conceived and designed the experiments; Analyzed and interpreted the data; Contributed reagents, materials, analysis tools or data.

### Funding statement

This work was supported by the 10.13039/501100003093Ministry of Higher Education, Malaysia (FRGS/1/2016/WAB09/UMT/02/2).

### Declaration of interests statement

The authors declare no conflict of interest.

### Additional information

No additional information is available for this paper.
